# Necrotizing Fasciitis Camouflaging As Cellulitis: A Case Report Highlighting Diagnostic Pitfalls

**DOI:** 10.7759/cureus.104734

**Published:** 2026-03-05

**Authors:** Sakshi Palav, Shravan Gangula, Jyothi R Patri

**Affiliations:** 1 College of Medicine, University of California, Berkeley, USA; 2 Family and Community Medicine, Coffeyville Regional Medical Center, Coffeyville, USA; 3 Family and Lifestyle Medicine, Heritage Valley Family Medicine Residency Program, Beaver Falls, USA

**Keywords:** laboratory risk indicator for necrotizing fasciitis (lrinec) score, necrotizing fasciitis management, necrotizing fasciitis mimic, necrotizing fasciitis misdiagnosis, necrotizing fasciitis (nf), necrotizing soft tissue infection (nsti), nf diagnostic modalities, nf differential diagnosis, nf mimicking cellulitis, nf suspicion index

## Abstract

Necrotizing fasciitis (NF) is a rapidly progressive, life-threatening soft tissue infection that is often misdiagnosed in its early stages due to clinical similarities with cellulitis. Early recognition and prompt surgical intervention are essential to reducing morbidity and mortality. This report presents one case of NF initially manifesting as cellulitis in a patient with multiple risk factors, including intravenous drug use and recent incarceration. Despite initial antibiotic therapy and transient clinical improvement, the patient experienced worsening pain and systemic symptoms. Imaging identified subcutaneous emphysema, leading to urgent surgical exploration that confirmed NF. This case highlights the importance of recognizing disproportionate pain, maintaining a high index of suspicion, and seeking early surgical consultation when NF is considered. The case was managed by family medicine residents and is presented to aid clinicians in recognizing and diagnosing NF among other potential conditions.

## Introduction

Necrotizing fasciitis (NF) is a rapidly progressing bacterial infection that destroys superficial fascia, subcutaneous tissue, and deep tissue. Bacterial entry typically occurs through a skin laceration, resulting in local pain, fever, systemic toxicity, tissue damage, and potentially fatal outcomes if not diagnosed and treated promptly [1]. As the infection advances, the skin may change from red and purple discoloration to patches of blue-gray. Eventually, the skin can break down into bullae, and cutaneous gangrene may develop. Tissue damage and systemic toxicity are caused by the release of cytokines and bacterial toxins, which trigger severe physiological responses [2].

Although the progression of NF is well documented, definitive diagnosis is achieved only through surgical debridement. Studies indicate that 70-85% of patients with NF are initially misdiagnosed due to symptoms that resemble those of other soft-tissue infections [3,4]. The clinical presentation, evaluation, and management of this patient are analyzed to demonstrate how NF can mimic other soft-tissue infections and to emphasize the importance of including it in the differential diagnosis. Despite advances in diagnostic imaging and laboratory testing, NF is frequently misdiagnosed in its early stages. This case illustrates key diagnostic pitfalls and highlights clinical features that should prompt early reconsideration of an initial diagnosis of cellulitis.

## Case presentation

**Figure 1:**
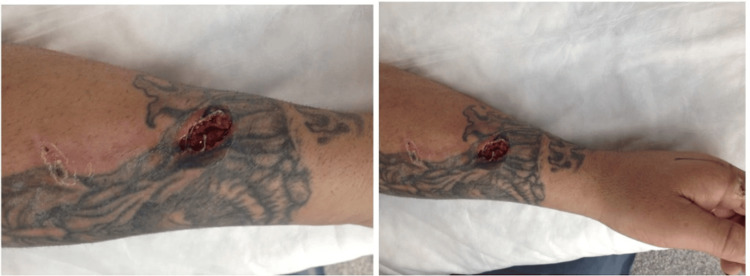
Clinical appearance of the left forearm lesion at presentation Close-up views of the left forearm demonstrate an open necrotic wound with surrounding erythema and soft-tissue destruction overlying a tattooed area. Written informed consent was obtained from the patient to publish identifying information.

A 33-year-old white male with a history of intravenous drug use, recent incarceration, and extensive upper extremity tattooing presented with progressive swelling, erythema, and pain of the left forearm. Initial examination demonstrated localized erythema and purulent drainage, consistent with cellulitis, and was treated with oral Bactrim by the jail nurse for 24 hours. Despite antibiotic management, the patient developed a temperature of 100.9°F with worsening symptoms and was transferred to the emergency department for further evaluation and management. Physical examination demonstrated an indurated and erythematous left forearm and a 1.5-cm opening on the distal forearm exuding foul-smelling discharge, as in Figure 1.

Range of motion in the left hand was limited, with increased temperature and fluctuating tenderness from the left cubital fossa to the hand. Initial laboratory evaluation revealed leukocytosis with neutrophilia, mild renal dysfunction, normal blood glucose (suggesting that hyperglycemia was not a contributing factor in this case), and hypocalcemia, consistent with a severe inflammatory process, as seen in Table 1.

**Table 1:** Patient laboratory investigations (baseline labs) Laboratory investigations demonstrate leukocytosis with neutrophilia, mild renal dysfunction, and hypocalcemia, consistent with a severe inflammatory process. Neutrophilia and leukocytosis are commonly observed in necrotizing fasciitis but are nonspecific and should be interpreted in the clinical context. BUN, blood urea nitrogen; WBC, white blood cells Source: [3]

Parameter	Patient value (mg/dL)	Normal range (mg/dL)	Clinical relevance
Comprehensive metabolic panel			
BUN	15	6 to 20	
Creatinine	1.18	0.6 to 1.3	Possibly early renal hypoperfusion
Sodium	134	135 to 145	
Potassium	3.8	3.7 to 5.2	
Chloride	102	96 to 106	
Bicarbonate	24	22 to 32	
Glucose	105	70 to 100	
Calcium	7.9	8.5 to 10.2	Associated with severe infection
Complete blood picture			
WBC	13.6 billion cells/L	3.4 to 9.6 billion cells/L	Reactive elevation with infection
Hemoglobin	12 grams/dL	13.2 to 16.6 grams/dL	
Hematocrit	35.8%	38.3% to 48.6%	
Platelets	178 billion cells/L	135 to 317 billion cells/L	
Neutrophils	80%	40% to 60%	Suggestive of bacterial infection

At this stage, the working diagnosis was cellulitis with abscess formation. Intravenous vancomycin (15 mg/kg every 12 hours) and Zosyn (3.375 g every six hours) were initiated to provide broad-spectrum antimicrobial coverage, and intravenous fluids were administered to address hemodynamic instability. Ibuprofen was given for analgesia. The patient initially responded favorably to treatment, with no reports of fever or severe pain.

**Figure 2:**
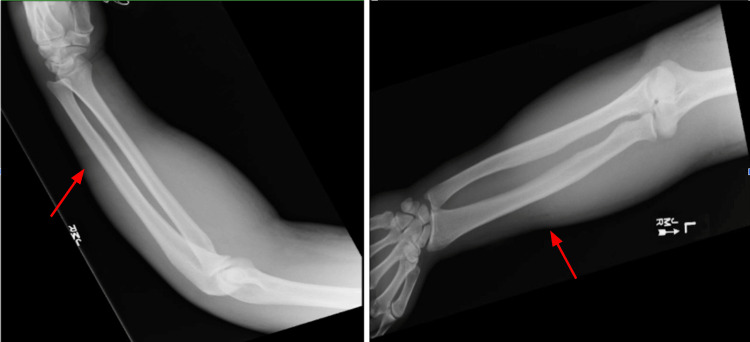
Plain radiographs of the left forearm Radiographs demonstrating subcutaneous emphysema (arrow), a key radiographic clue supporting necrotizing fasciitis.

Twelve hours after admission, the patient experienced worsening induration despite an improved white blood cell count of 9.7 and reported pain disproportionate to the clinical findings. The severity of the pain relative to the physical findings raised concern about a deeper soft-tissue infection. Plain radiography of the left forearm demonstrated extensive soft tissue swelling with subcutaneous emphysema, a finding highly suggestive of NF in the appropriate clinical context (Figure 2).

**Figure 3:**
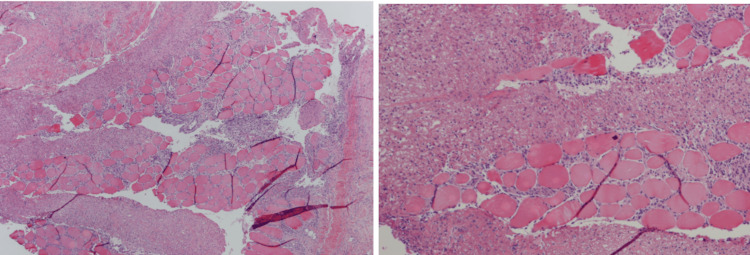
Histopathological findings from surgical debridement Histopathological features of NF. Hematoxylin and eosin stain sections showing necrotic skeletal muscle fibers and infiltration by dense, acute inflammatory infiltrate (original magnification ×10, 20). NF, necrotizing fasciitis

These factors, along with preliminary wound cultures positive for gram-positive cocci, gram-positive bacilli, and gram-negative bacilli, raised a high index of suspicion for NF Type I infection. The patient was taken emergently to the operating room, where surgical exploration confirmed NF by frozen section (Figure 3).

Final wound cultures were positive for *Streptococcus* species, *Peptostreptococcus*, and *Prevotella intermedia*. Following infectious disease consultation, Zosyn was discontinued, and vancomycin (15 mg/kg every 12 h IV) and Unasyn (3 g every six hours IV) were continued. Due to the patient's history of substance use disorder, patient-controlled analgesia with Dilaudid was used for pain management. The patient was gradually weaned and transitioned to oral Norco with adjunct gabapentin.

**Figure 4:**
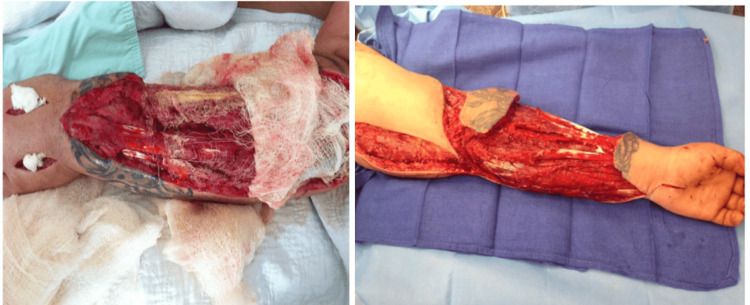
Forearm after debridement Forearm after debridement on day five (left) and day 15 (right).

Extensive debridement was performed, followed by multiple subsequent surgical interventions. After the procedure, a wound vacuum-assisted closure device was applied, and the patient was transferred to the intensive care unit, where hemodynamic status improved. Serial debridement and irrigation were performed on days 1, 3, 5, and 8, with additional debridement, irrigation, and Integra placement on day 11. By day 15, the infection had resolved, and the patient's wound was closed via split-thickness skin grafting reconstruction. Ultimately, broad-spectrum antibiotic therapy, debridement, and reconstruction led to a favorable clinical outcome, and the patient subsequently returned to the correctional facility with a good functional recovery (Figure 4).

A structured timeline of the patient’s clinical course, including symptom progression, diagnostic evaluation, and therapeutic interventions, is summarized in Table 2.

**Table 2:** Timeline of patient symptoms, diagnosis, and treatment ED, emergency department; CT, computed tomography; IV, intravenous; NF, necrotizing fasciitis; ICU, intensive care unit; STSG, split-thickness skin graft; PICC, peripherally inserted central catheter

Day/hours of case	Symptoms, findings, and treatment performed
Day 0 (0 hours)	Onset of pain and swelling
Day 1	Fever and worsening erythema, ED presentation
Day 1.5 (36 hours)	CT imaging performed, broad-spectrum IV antibiotics initiated
Day 2	Surgical consultation, irrigation, and debridement × 1 and NF confirmed
Day 4	Second-look debridement, ICU monitoring
Day 6	Irrigation and debridement × 3, clinical stabilization
Day 9	Irrigation and debridement × 4, transition to targeted antibiotics
Day 11	Irrigation and debridement × 5 with Integra placement
Day 15	STSG reconstruction performed
Day 16	Infection resolved, PICC line taken out, patient released with Integra in place, and plastic surgery follow-up in four to six weeks

## Discussion

NF is a rapidly progressing bacterial infection that targets the subdermal fascial planes, leading to destruction of superficial fascia, subcutaneous tissue, and deep tissue. The annual incidence in the USA is between 500 and 1,500 cases reported, with mortality rates for cases receiving treatment ranging from 6% to 76%, but nearing 100% for cases without treatment [5]. Literature suggests that surgical intervention beyond 24 hours from diagnosis is directly correlated to increased mortality, and failure to diagnose promptly can result in death [6].

NF is classified into two types. Type I is polymicrobial, caused by a combination of gram-negative and anaerobic bacteria, whereas Type II is monomicrobial and typically results from a single pathogen, such as group A *Staphylococcus aureus* or *Streptococcus*, in single-site infections. Type I is generally associated with enteric pathogens and occurs in patients with diabetes, peripheral vascular disease, recent surgery, or immunocompromised states. Type II infections are due to skin flora and can affect otherwise healthy individuals of any age. In both types, the infection initially travels along the fascial plane, sparing surrounding tissues and making early diagnosis challenging. As the infection progresses, it rapidly spreads to the fascia and perifascial planes, resulting in secondary infection of the skin, soft tissues, and muscles [7].

Clinical manifestations of NF may be acute or sub-acute. Affected areas are typically erythematous, swollen, warm, shiny, and excessively tender. Patients often experience pain that is disproportionate to clinical findings, and the skin may progress from red and purple discoloration to patches of blue and gray. As the disease advances, bullae develop, and ultimately, frank cutaneous gangrene occurs [8].

Several factors contributed to the delayed recognition of NF in this case. The patient’s NF was initially misdiagnosed as cellulitis, likely due to the presence of an open wound on the left distal forearm and the erythematous, indurated appearance, which are consistent with cellulitis, especially in the context of intravenous heroin use. Extensive tattooing obscured early cutaneous changes, while transient initial improvement with antibiotics provided false reassurance. While both cellulitis and NF can cause necrosis, cellulitis is confined to the skin, whereas NF extends into deeper tissues [9]. Although cellulitis is more common, NF must be considered when pain is disproportionate to physical findings or when clinical progression is rapid. Table 3 outlines key distinctions between these two conditions.

**Table 3:** Key clinical features distinguishing NF from cellulitis NF, necrotizing fasciitis Source: [9]

Feature	Cellulitis	Necrotizing fasciitis
Pain	Proportional	Out of proportion
Progression	Over days (gradual)	Within a few hours (rapid)
Systemic toxicity	Mild	Severe
CT imaging	No gas	Subcutaneous emphysema
Surgical findings	Not required	Diagnostic

This case underscores the importance of thorough clinical examination. As most cases are initially misdiagnosed, clinicians should include NF in the differential diagnosis when patients present with signs of acute or sub-acute infections such as cellulitis, abscess, necrotizing myositis, gas gangrene, deep venous thrombosis, septic arthritis, warfarin-induced skin necrosis, brown recluse spider bite, and gangrene with secondary infection. Because many of these conditions are soft-tissue infections, clinicians should maintain a high index of suspicion for NF in similar presentations. When clinical suspicion is high, prompt biopsy is necessary to confirm the diagnosis and facilitate timely debridement, thereby reducing morbidity and mortality.

Diagnosis of NF relies on clinical and laboratory findings that prompt surgical exploration, with tissue biopsy serving as the definitive diagnostic test. Imaging modalities such as X-rays, ultrasound, CT, and MRI can assist in revealing asymmetrical fascial thickening, soft-tissue air, blurring of fascial planes, inflammatory fat stranding, reactive lymphadenopathy, and nonenhancement of muscular fascia with biopsy findings revealing extensive tissue destruction, vascular thrombosis, and infiltration of fascial planes with inflammatory cells and bacteria [9,10]. The Laboratory Risk Indicator for Necrotizing Fasciitis (LRINEC) score differentiates NF from other soft-tissue infections using CRP, WBC, hemoglobin, sodium, creatinine, and glucose levels; however, waiting for laboratory results may not be feasible in acute settings [11].

Wei et al. (2023) discussed in detail the diagnostic modalities used for NF evaluation and management [12], as shown in Table 4.

**Table 4:** Diagnostic modalities for NF Summary of the diagnostic modalities utilized for the evaluation and management of NF. LRINEC, laboratory risk indicator for necrotizing fasciitis; SIARI, site, immunosuppression, age, renal failure, and inflammatory markers Source: [12]

Diagnostic modality	Salient features	Strengths	Weaknesses
Ultrasound	Fascial thickening, fluid collections, early gas	Bedside, no radiation, early clue to gas	Operator-dependent, limited depth, incomplete fascial view
Plain radiography (X-ray)	Subcutaneous gas, soft tissue swelling, foreign bodies	Fast, inexpensive, widely available	Low sensitivity, often normal early
CT	Gas along fascial planes, fluid tracking, soft tissue changes	Rapid, excellent detail, reliable for gas	Radiation, contrast limits in renal disease
MRI	Diffuse fascial edema, clear tissue plane contrast	Most sensitive for soft tissue involvement	Expensive, slow, contraindications (implants, claustrophobic)
LRINEC	Integrates CRP and routine labs into a risk score	Quick risk stratification from routine labs	False negatives, cannot replace clinical judgment
SIARI	Combines epidemiology + labs for risk estimates	Emerging alternative with promising diagnostic performance	Limited validation, adjunct only

Despite advancements in imaging modalities, clinical diagnosis remains essential for the timely identification of NF. While scoring systems and imaging can support clinical judgment, definitive diagnosis requires surgical exploration. Early multidisciplinary involvement and prompt operative management are critical to improving outcomes. As intraoperative biopsy is the only definitive diagnostic modality, clinicians should not delay intervention in favor of imaging or other diagnostic methods. This highlights the importance of clinical acumen in promptly recognizing symptoms suggestive of NF, even when diagnostic methods are inconclusive. Yu et al. describe clinical modalities for initial evaluation of NF based on clinical signs and symptoms, as shown in Table 5 [13].

**Table 5:** Clinical modalities for NF evaluation as per Yu et al. (2022) NF, necrotizing fasciitis Source: [13]

Variable	Clinical manifestations
Skin	Redness, swelling, tenderness, erythema, increasing temperature of the area, skin discoloration to purple, blue, and black, bullae, edema, skin necrosis, crepitus
Pain	Out of proportion, severe at presentation
Physical	Nausea, fever, diarrhea, dizziness

Management of NF requires aggressive surgical debridement of all necrotic tissue. Early operative intervention within 12 hours remains the cornerstone of improved survival in NF [6]. Debridement should be performed daily or at regular intervals until healthy, viable, bleeding tissue is observed. Empiric broad-spectrum antibiotics are initiated and subsequently tailored to target specific bacterial organisms once cultures are available [14]. In severe cases involving gas-forming organisms, intravenous immunoglobulin (IVIG) therapy may be considered [15].

## Conclusions

NF is a life-threatening condition that can initially resemble cellulitis, leading to diagnostic delays, particularly when tattoos or skin alterations obscure early clinical signs. Prompt surgical consultation upon suspicion of NF is essential to facilitate timely debridement and reduce morbidity and mortality. This case highlights the significance of recognizing disproportionate pain as an early warning sign, monitoring for rapid clinical progression, and identifying imaging features such as subcutaneous emphysema on plain radiography that suggest deep soft-tissue infection.
